# Higher Cortisol Predicts Less Improvement in Verbal Memory Performance after Cardiac Rehabilitation in Patients with Coronary Artery Disease

**DOI:** 10.1155/2013/340342

**Published:** 2013-01-16

**Authors:** Mahwesh Saleem, Nathan Herrmann, Walter Swardfager, Paul I. Oh, Prathiba Shammi, Gideon Koren, Stan Van Uum, Alexander Kiss, Krista L. Lanctôt

**Affiliations:** ^1^Neuropsychopharmacology Research Group, Sunnybrook Health Sciences Centre, 2075 Bayview Avenue, Toronto, ON, Canada M4N 3M5; ^2^Department of Pharmacology & Toxicology, University of Toronto, 1 King's College Circle, Toronto, ON, Canada M5S 1A8; ^3^Department of Psychiatry, University of Toronto, 1 King's College Circle, Toronto, ON, Canada M5S 1A8; ^4^Cardiac Rehabilitation and Secondary Prevention Program, Toronto Rehabilitation Institute, 345 Rumsey Road, Toronto, ON, Canada M4G 1R7; ^5^Division of Clinical Pharmacology, Sunnybrook Health Sciences Centre, 2075 Bayview Avenue, Toronto, ON, Canada M4N 3M5; ^6^Neuropsychology, Sunnybrook Health Sciences Centre, 2075 Bayview Avenue, Toronto, ON, Canada M4N 3M5; ^7^Motherisk Program, The Hospital for Sick Children, 555 University Avenue, Toronto, ON, Canada M5G 1X8; ^8^Department of Medicine, University of Western Ontario, St. Joseph's Health Care, 268 Grosvenor Street, London, ON, Canada N6A 4V2; ^9^Clinical Epidemiology, Sunnybrook Health Sciences Centre, 2075 Bayview Avenue, Toronto, ON, Canada M4N 3M5

## Abstract

*Objective*. While physical activity can improve verbal memory performance in subjects with coronary artery disease (CAD), there is large variability in response. Elevated cortisol production has been suggested to negatively affect verbal memory performance, yet cortisol concentrations have not been assessed as a predictor of response to exercise intervention in those with CAD. *Methods*. CAD patients participating in a one-year cardiac rehabilitation program were recruited. Memory was assessed with the California Verbal Learning Test second edition at baseline and one year. Cortisol was measured from a 20 mg, 3.0 cm hair sample collected at baseline. *Results*. In patients with CAD (*n* = 56, mean ± SD age = 66 ± 11, 86% male), higher cortisol (hair cortisol concentrations ≥ 153.2 ng/g) significantly predicted less memory improvement (F_1,50_ = 5.50, *P* = 0.02) when controlling for age (F_1,50_ = 0.17, *P* = 0.68), gender (F_1,50_ = 2.51, *P* = 0.12), maximal oxygen uptake (F_1,50_ = 1.88, *P* = 0.18), and body mass index (F_1,50_ = 3.25, *P* = 0.08). *Conclusion*. Prolonged hypothalamic pituitary adrenal axis activation may interfere with exercise-related improvements in memory in CAD.

## 1. Introduction

Coronary artery disease (CAD) is associated with an increased rate of decline in verbal memory [1]. For those with CAD, cognitive performance represents a significant determinant of long-term quality of life [2] as exemplified by associations with poor outcomes including unemployment, mortality, and failure to comply with risk factor management [2–4]. However, the mechanisms underlying cognitive changes in CAD are incompletely understood.

 Epidemiological studies show that cardiopulmonary fitness is associated with better cognitive performance in older subjects [5], particularly in those with CAD [6]. Exercise interventions, such as cardiac rehabilitation (CR), can improve cognitive performance, although interindividual responses to exercise training can vary [7].

 CAD and related cardiovascular risk factors have also been associated with higher cortisol concentrations [8]. While prolonged elevations in cortisol have been associated with memory impairment [9], dysregulation of the hypothalamic pituitary adrenal (HPA) axis has not been probed as a predictor of cognitive response to exercise in this population. The objective of the present study was to assess the relationship between baseline hair cortisol concentrations and change in verbal memory performance in subjects with CAD undertaking CR. Hair cortisol has been suggested to reflect longer term cortisol secretion [10, 11] rather than state concentrations, suggesting a potential clinically useful marker. We hypothesized that cortisol concentrations measured from hair would be associated with less improvement in verbal memory performance over one year.

## 2. Methods

### 2.1. Subject Screening and Recruitment

This study was approved by the local Research Ethics Boards. We screened consecutive patients entering the one-year CR program of the Toronto Rehab between May 2007 and June 2009 who agreed to be contacted for research. Patients with sufficient evidence of a cardiac diagnosis in their medical history and who completed a cardiopulmonary fitness test were eligible for the study. Eligible subjects who provided written informed consent were further assessed for inclusion/exclusion criteria. We included patients if they had evidence of CAD based on previous hospitalization for acute myocardial infarction (MI), coronary angiographic evidence of ≥50% blockage in one or more major coronary arteries, or a prior revascularization procedure a minimum of 6 weeks after a MI or coronary artery bypass graft surgery (CABG) or 3 weeks since a percutaneous coronary intervention (PCI). We excluded patients based on previously diagnosed neurodegenerative illness or premorbid psychiatric diagnoses other than depression, any illnesses affecting the HPA axis, and the use of drugs affecting the HPA axis or cognition. We screened for cognitive status using the Mini Mental Status Examination (MMSE) and excluded patients with MMSE <24. We also excluded subjects if sufficient hair (3.0 cm) for cortisol analysis could not be provided.

### 2.2. CR Protocol

The CR program at the Toronto Rehab is an exercise intervention program consisting of both aerobic and resistance training in a group setting under the supervision of exercise and medical specialists. Patients received short lectures on the risk factors associated with CAD and the value of exercise and attended supervised exercise visits that included an aerobic walk or walk/jog once per week for 36 weeks and once per month for the remaining three months of the year. Patients were also expected to exercise five out of seven days of the week at home and document the duration, intensity, and frequency of the exercise in weekly exercise diaries, which were monitored by an assigned exercise supervisor. Depression and cardiopulmonary fitness assessments were carried out at entry and one-year time points. Attendance at weekly visits was recorded and completion status was determined based on the case-manager's assessment of compliance with attendance at scheduled CR sessions, individually prescribed exercise in between the scheduled CR sessions and completion of cardiopulmonary fitness tests. For this study, patients were recruited at entry to the CR program and followed over the standard CR protocol. Hair for cortisol assays was taken at baseline. Cognitive and psychosocial outcomes were measured at entry and at the end of CR. 

### 2.3. Subject Demographics

Demographic and clinical characteristics, as well as a detailed medical history including comorbidities independent of CAD were collected from patient interviews. We also collected cardiac medical history, concomitant medications and cardiac health indicators including heart rate, blood pressure, body mass index (BMI), height, body mass, maximal oxygen uptake, the most reliable and reproducible measure of cardiopulmonary fitness [12], lipids, and percentage of body fat from patient charts at the Toronto Rehab cardiac program.

### 2.4. Verbal Memory Assessment

We assessed verbal memory using the California Verbal Learning Test second edition (CVLT-II), which yields multiple measures of learning, immediate recall, and delayed verbal recall. The CVLT-II is recommended by the National Institute of Neurological Disorders and Stroke and Canadian Stroke Network harmonized standards for the investigation of vascular cognitive impairment, and it is sufficiently sensitive to capture clinically meaningful variation in cognitive performance in the study population [4]. A trained researcher administered the CVLT-II at a standardized time (0930 hr ± 30 min) under the supervision of an experienced clinical neuropsychologist.

The CVLT-II word list includes 16 words that fall into four different categories. This list of words is read to the subject and then recalled orally by the subject. This procedure is carried out five times constituting the five learning trials, which are a measure of learning. A distractor list is then read to the subject and recalled orally by the subject, after which the subject is prompted to recall the original list. This is defined as the short-delay free recall (SDFR). After 20 minutes, the subject is prompted to recall the original word list again, which is defined as long-delay free recall (LDFR). *Z*-scores for SDFR and LDFR were computed from age- and gender-matched norms, and a composite measure of verbal recall was obtained by summing the two measures as has been done previously [6]. A higher *Z*-score reflects better performance on the test as *Z*-scores follow a normal distribution. This allows individual *Z*-scores to be quantitatively combined to generate a composite score [13].

### 2.5. Psychosocial Assessments

We administered the Structured Clinical Interview for Depression at baseline to determine whether subjects met the Diagnostic and Statistical Manual 4th edition depression criteria. Depression is associated with increased mortality in subjects with CAD [14] and with poorer cognitive performance in older subjects [15]. A clinically experienced psychiatrist conducted researcher training and quality assurance for interview skills and diagnostic accuracy and reviewed results. 

We assessed subjective stress at baseline and one year using the 10-item perceived stress scale (PSS), which measures the degree to which life events are appraised as stressful over the previous month on a scale of 0–40 [16]. We calculated average PSS scores over one year to obtain a longitudinal measure of perceived stress.

### 2.6. Cortisol Measurement

Previous studies measuring cortisol concentrations may have been hampered by a lack of a simple method to determine long-term cortisol production. This study used a relatively new method, determining the cortisol content of hair as a measure of longer-term cortisol secretion. This method of cortisol measurement is noninvasive, not affected by the intra- and interday variation resulting from factors such as diurnal rhythmicity, and does not impact cortisol production in response to sampling or testing [17]. Sauvé et al. [11] showed that hair cortisol concentrations were significantly associated with 24-hour urine cortisol concentrations supporting the relevance of hair cortisol measurement as a biomarker of longer-term exposure. To date, higher hair cortisol has been indicated as a potential biomarker of chronic stress in neonates [18], patients with chronic pain [19], Cushing's syndrome [10], alcohol withdrawal [20], unemployed individuals [21], pregnant women in their third trimester [22], and prior to a MI [8]. In the present study, we measured cortisol concentrations from 20 mg of 3.0 cm long hair samples taken at baseline to determine mean cortisol secretion over the previous three months. 

### 2.7. Hair Collection

Hair samples from patients were collected from the posterior vertex region in accordance with the consensus statement from the Society of Hair Testing [23]. The hair sample consisted of 20 mg of hair (approximately 100–150 strands). The sample was collected as close to the root as possible to obtain the most recent cortisol deposition. Based on an estimate of 1.0 cm of growth each month [24], we used the 3.0 cm of hair proximal to the root to assay cortisol deposition over the past three months. The hair sample was stored at room temperature until analysis. 

### 2.8. Cortisol Assay

The hair sample was finely chopped with surgical scissors and shaken at 100 RPM for 16 hours at 50°C in 1 mL of methanol. Methanol extractions were evaporated in a dry bath under a stream of nitrogen gas at 50°C. The sample was reconstituted in a phosphate-buffered saline solution (to 250 *μ*L, pH-8.0). The sample was vortexed and 50 *μ*L of phosphate-buffered saline was added to the plate wells in duplicate. Cortisol measurements were performed using a salivary enzyme-linked immunosorbent assay (ELISA; Alpco Diagnostics, NH, USA) as described previously [19]. Positive and negative controls were included. The negative control, which contained buffer only, was used to determine any nonspecific binding, which was subtracted from all other values before interpretation. The intra-assay coefficient of variation, which was 3.8% (*n* = 5), and the interassay coefficient of variation, which was 8% (*n* = 6), were obtained using a standard hair sample. The limit of detection of the ELISA cortisol kit was 1.14 ng/mL (Alpco Diagnostics) [8]. Cross-reactivity of other steroids with the ELISA kit antibodies was reported as follows: corticosterone 31%, progesterone < 2%, deoxycortisol < 2%, dexamethasone < 2%, estriol, estrone and testosterone < 0.001%, and cortisone 1%. Patients were dichotomized into normal and high cortisol groups for analysis since cortisol concentrations are skewed [25]. A reference range has been established for hair cortisol concentrations, suggesting a median of 46.1 ng/g with a range of 17.7 to 153.2 ng/g [11]. We classified CAD patients with hair cortisol concentrations within the reference range as having normal cortisol and patients above or equal to 153.2 ng/g as having high cortisol. Samples with cortisol concentrations above 1500 ng/g were excluded from the analysis since these concentrations indicate supraphysiological levels, possibly due to Cushing's syndrome or contamination from hydrocortisone creams or ointments [10].

### 2.9. Statistical Analyses

All analyses were performed using SPSS statistical software (version 18.0, SPSS Inc., Chicago, IL, USA) and results were considered significant at a two-tailed *P* ≤ 0.05. Differences in baseline sociodemographic characteristics, cardiac risk factors, concomitant medication use, and medical comorbidities between normal and high cortisol groups were assessed by Pearson's *χ* ^2^, or univariate analyses of variance as appropriate. Repeated measures general linear models were used to assess an intrasubject time × cortisol group interaction in predicting composite verbal memory *Z*-scores over one year. We selected age [26], gender [27], maximal oxygen uptake [28], and BMI [29] as covariates *a priori*. Depression and average PSS scores over one year were included in additional models as covariates in order to assess the contribution of cortisol to cognitive outcomes independent of these variables. Variables that were significantly different between the normal and high cortisol groups were also included in additional *post-hoc* analyses.

## 3. Results

### 3.1. Subject Characteristics

**Figure 1:**
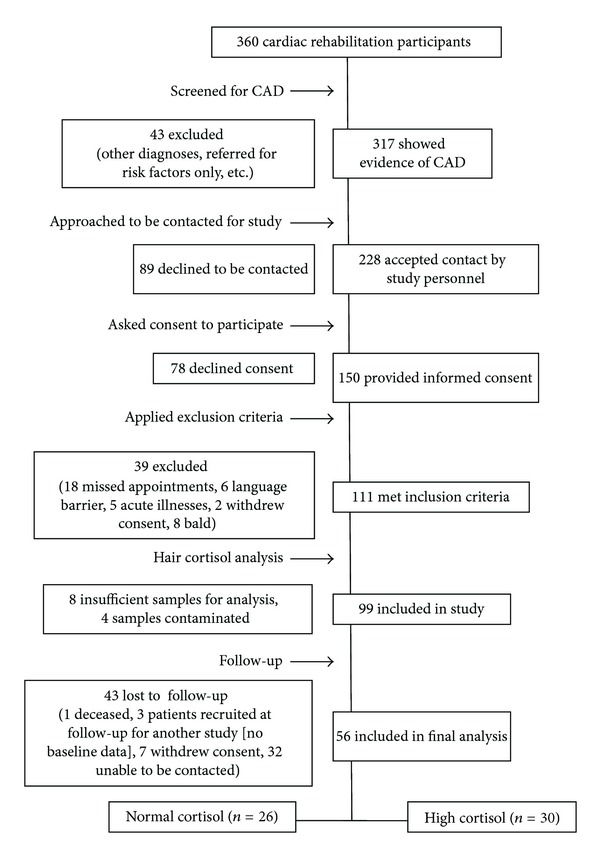
Patient inclusion process for the study.

The sample was composed of 56 subjects who completed the one-year CR program (Figure 1). Although data on those who declined to participate in the study were unavailable, characteristics of the study participants were similar to a large unselected sample from the center database (*n* = 366) in terms of age (66 ± 11 versus 64 ± 11 years), gender (86% versus 76% male), cardiac diagnoses (e.g., PCI 50% versus 48%; CABG 38% versus 37%), and vascular risk factors (e.g., BMI 27.3 ± 4.2 versus 28.0 ± 4.7) [30]. Included subjects were also similar in demographics (age = 66 ± 11 years, 86% male, *n* = 56) to those who were lost to follow-up (age = 61 ± 11 years, 70% male, *n* = 43). Patients lost to follow-up had significantly lower baseline cortisol concentrations (F_1,98_ = 6.93, *P* = 0.01) compared to those included in the analysis. There were no differences in baseline cognitive scores between those lost to follow-up and those who completed the one-year CR program.

Twenty-six patients showed normal (median cortisol concentration (interquartile range) = 111.4 ng/g (27.4 ng/g)) and 30 patients showed high (262.8 ng/g (276.0 ng/g)) cortisol concentrations. All patients had hypercholesterolemia and were taking statins. There were no differences in sociodemographic characteristics, cardiac risk factors, BMI, concomitant medication use, or medical comorbidities between normal and high cortisol groups except that a higher proportion of patients in the high cortisol group had a history of CABG (Table 1). Completion of CR (*χ* ^2^ = 0.06, *P* = 0.80) and the number of CR sessions attended (F_1,55_ = 0.41, *P* = 0.53) did not differ between the normal and high cortisol groups.

### 3.2. Associations between Cortisol and Cognitive Performance

14.3% of patients performed 1.5 SD below the expected norm at baseline; however, mean SDFR and LDFR outcomes of the CVLT-II improved over one year as shown in Table 2. Patients in both the normal and high cortisol groups showed significant improvement in composite memory *Z*-scores scores over one year (Table 2).

**Figure 2:**
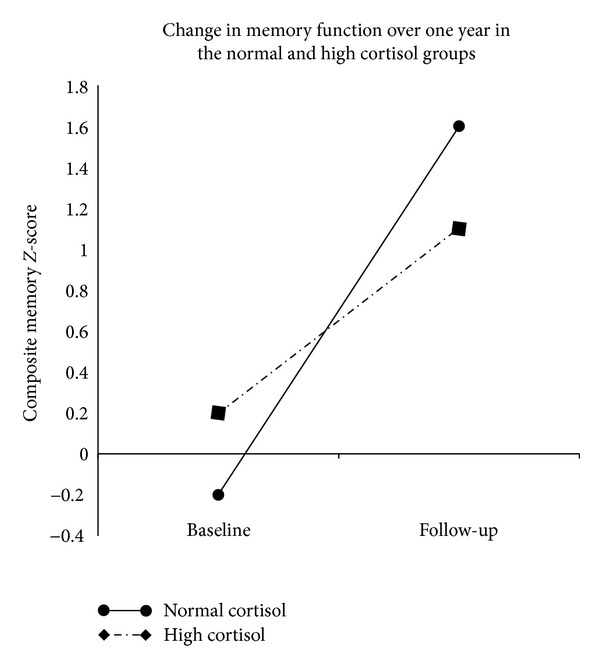
Change in verbal memory performance over one year in the normal and high cortisol groups. In a repeated measures model, significant intrasubject effects (F_1,50_ = 5.50, *P* = 0.02) indicate less improvement in verbal memory performance over one year in the high cortisol group (dashed line) compared to the normal cortisol group (solid line). No significant intersubject effects were found.

In a repeated measures general linear model predicting composite verbal memory *Z*-scores controlling for age, gender, maximal oxygen uptake, and BMI, we observed a significant intrasubject time × cortisol group interaction (F_1,50_ = 5.50, *P* = 0.02) such that patients in the high cortisol group showed less improvement (Figure 2). Age (F_1,50_ = 0.17, *P* = 0.68), gender (F_1,50_ = 2.51, *P* = 0.120), maximal oxygen uptake (F_1,50_ = 1.88, *P* = 0.18), and BMI (F_1,50_ = 3.25, *P* = 0.08) were not significant predictors. 

### 3.3. *Post-Hoc* Analyses

Depression (F_1,50_ = 0.07, *P* = 0.79) and average PSS scores over one year (F_1,50_ = 0.13, *P* = 0.72) were not significant predictors of change in memory performance. Adding depression and average PSS scores to the model did not change the associations between higher cortisol concentrations and less improvement in verbal memory performance (F_1,50_ = 5.35, *P* = 0.03 and F_1,50_ = 5.26, *P* = 0.03, resp.). 

CAD patients who had undergone CABG surgery had higher mean cortisol concentrations compared to those who had not (Table 1). When added to the model, CABG was not associated with change in memory performance (F_1,50_ = 0.50, *P* = 0.48) and a trend remained for higher cortisol predicting less improvement in verbal memory performance (F_1,50_ = 3.83, *P* = 0.06).

Baseline fitness was explored as a potential moderator to explain the variation in the effect of cortisol on memory change since fitness has been independently associated with cognitive performance in this population [6]. A baseline maximal oxygen uptake × cortisol group interaction was entered into the original model in addition to gender, maximal oxygen uptake, and BMI. In this model, baseline BMI (F_1,50_ = 5.04, *P* = 0.03), cortisol group (F_1,50_ = 7.44, *P* < 0.01), and the maximal oxygen uptake × cortisol group interaction (F_1,50_ = 5.03, *P* = 0.03) were significant predictors of change in verbal memory *Z*-scores over one year. The maximal oxygen uptake × cortisol group interaction term indicated that higher fitness predicted greater improvement in verbal memory performance in the high cortisol group but not in the normal cortisol group. Gender (F_1,50_ = 3.68, *P* = 0.06) and maximal oxygen uptake (F_1,50_ = 2.33, *P* = 0.13) were not significant predictors. 

## 4. Discussion

The present study is the first to examine the association between baseline deposition of cortisol in hair and change in memory performance in CAD patients undertaking exercise. We saw an improvement in verbal memory performance after exercise, consistent with previous findings [5], but patients with higher baseline hair cortisol secretion showed less improvement over one year of CR. The association between higher cortisol and less verbal memory improvement persisted even when controlling for depression and perceived stress.

Hair cortisol concentrations in the high cortisol group of the present study (262.8 ng/g (276.0 ng/g)) were comparable to those reported previously (median (full range) = 295.3 ng/g (105.4–809.3 ng/g)) during hospital admission for acute MI [8]. Patients included in the present study were referred to the CR program at least six weeks after MI, which may account for a proportion of patients with lower cortisol concentrations in the present study. 

A large body of evidence shows associations between higher cortisol concentrations and both atrophy of the hippocampus, the brain region subserving verbal memory [31, 32], and memory impairment [33]. Exposure to stress hormones can lead to a reduction in neuronal health and survival, especially in the hippocampus [34]. In response to chronic stress, neurons undergo morphological changes, which have a deleterious impact on brain plasticity [35] and adult hippocampal neurogenesis [36]. Animal studies have shown that cortisol can decrease the availability of neurotrophic factors such as the brain-derived neurotrophic factor in the hippocampus [36]. The present finding that high cortisol attenuated improvements in memory scores over the course of a one-year exercise intervention would be consistent with this suggestion.

In a *post-hoc* analysis, we explored CABG as an additional covariate since the incidence of CABG was significantly different between the normal and high cortisol groups. Despite existing evidence of cognitive changes after CABG [37], CABG was not a significant predictor of change in memory performance in this study. Some studies have implicated irregularities in HPA axis function, specifically alterations in the cortisol negative feedback mechanism, in postoperative cognitive deficits [38, 39], and this would be consistent with the persistence of a trend for higher cortisol predicting changes in memory performance even when controlling for CABG. 

An additional *post-hoc* analysis suggested that the relationship between cortisol and change in verbal memory performance might be modified by cardiopulmonary fitness at baseline; subjects with higher cortisol and poorer fitness entering CR improved significantly less. Further investigations are needed to clarify the potentially bidirectional relationships between physical activity and cortisol concentrations and their effects on cognition.

This study was strengthened by a prospective design, by the use of a noninvasive measure of long-term cortisol and by the use of a highly sensitive instrument to assess verbal memory. This study was limited by a small sample size, restricting the number of covariates; however, no differences in possible confounders between cortisol groups were detected, with the exception of surgical history, which was not associated with verbal memory changes in this population. Practice effects may have contributed to the overall improvement in verbal memory; however, a one-year interval between testing would be expected to minimize such effects on tests of verbal learning and memory [40]. Furthermore, normal and high cortisol groups were equally subject to possible practice effects. Data were not available on those who declined to participate in the study to assess recruitment bias beyond comparison with an unselected sample. Possible selection biases associated with CR referral, study recruitment, and CR completion might further limit the generalizability of the results. Some patients were unavailable for follow-up cardiopulmonary testing precluding longitudinal fitness assessment. Changes in maximal oxygen uptake may have offered further insight into the effects of cortisol on the contribution of exercise to cognitive changes. Future studies might examine the impact of exercise on cortisol concentrations.

## 5. Conclusions

 Higher baseline cortisol concentrations predicted less improvement in verbal memory performance over the course of CR. Chronic HPA axis activation might limit the beneficial effects of exercise training on cognitive performance in patients with CAD. 

## Figures and Tables

**Table 1:** Subject demographics and clinical characteristics (*n* = 56)^1^.

	Normal cortisol (*n* = 26)	High cortisol (*n* = 30)	Statistic (*F* or *χ* ^2^)	*P* value^2^ (*P* ≤ 0.05)*
Sociodemographics				
Age, years, mean (SD)	67 (12)	65 (11)	0.62	0.43
Gender, % male (*n*)	92.3 (24)	80 (24)	1.72	0.19
Marital status, % married (*n*)	88.5 (23)	73.3 (22)	1.62	0.20
Ethnicity, % Caucasian (*n*)	84.6 (22)	93.3 (28)	1.02	0.31
Employment, % employed (*n*)	42.3 (11)	30 (9)	0.03	0.86
Total education, years, mean (SD)	16.5 (3)	17 (3.5)	0.59	0.45
Time since acute coronary event (wks)	21.3 (40.9)	28.4 (77.9)	0.18	0.68
Vascular risk factors, % (*n*)				
Hypertension	53.8 (14)	63.3 (19)	0.52	0.47
Hyperlipidemia	100 (26)	100 (30)	—	—
Diabetes	7.7 (2)	16.7 (5)	1.03	0.31
BMI, kg/m^2^, mean (SD)	27.1 (5.0)	27.4 (3.5)	0.10	0.75
Waist, cm, mean (SD)	96.2 (10.1)	98.8 (8.8)	1.13	0.29
Cardiac history, % (*n*)				
PCI	57.7 (15)	43.3 (13)	1.15	0.28
CABG	23.1 (6)	50 (15)	4.31	0.04*
MI	57.7 (15)	40 (12)	1.75	0.19
Cardiopulmonary fitness parameters				
Maximum heart rate, bpm, mean (SD)	118.0 (18.1)	121.2 (21.7)	0.36	0.55
Maximum systolic blood pressure, mm Hg, mean (SD)	175.7 (23.5)	176.6 (27.0)	0.02	0.90
Maximum diastolic blood pressure, mm Hg, mean (SD)	76.2 (9.0)	79.9 (11.1)	1.86	0.18
Maximal oxygen uptake, mL/kg/min, mean (SD)	19.7 (4.5)	19.6 (4.6)	0.001	0.98
Concomitant medications, % (*n*)				
Beta-blocker	80.8 (21)	70 (21)	0.86	0.35
Calcium channel blocker	26.9 (7)	10 (3)	2.72	0.10
Diuretics	23.1 (6)	20 (6)	0.08	0.78
Antihypertensives	50 (13)	63.3 (19)	1.01	0.32
Statins	100 (26)	100 (30)	—	—
Antidiabetics	3.8 (1)	16.7 (5)	2.39	0.12
Antidepressants	3.8 (1)	10 (3)	0.80	0.37
Anxiolytics	11.5 (3)	3.3 (1)	1.41	0.23
Psychosocial assessments				
PSS, average over one year (SD)	19.8 (11.4)	18.6 (10.7)	0.16	0.69
Depression, % (*n*)	26.9 (7)	30 (9)	0.07	0.80

^1^PCI: percutaneous coronary intervention; CABG: coronary artery bypass graft; MI: myocardial infarction; bpm: beats per minute; PSS: perceived stress scale.

^2^Two-tailed significance in Pearsons *χ* ^2^ or one-way ANOVA.

**Table 2:** CVLT-II outcomes and composite memory *Z*-scores at baseline and at the end of CR^1^.

Cortisol groups	CVLT-II outcomes	Raw scores		*Z*-scores		
		Baseline Mean (SD)	Follow-up Mean (SD)	Baseline Mean (SD)	Follow-up Mean (SD)	*P* value^2^ (*P* ≤ 0.05)*
	SDFR	8 (3)	10 (3)	−0.06 (1.02)	0.81 (1.06)	<0.00005*
Normal cortisol	LDFR	8 (3)	11 (3)	−0.17 (1.06)	0.75 (1.01)	<0.00005*
	Composite memory	—	—	−0.23 (2.00)	1.56 (1.96)	<0.00005*

High cortisol	SDFR	9 (4)	10 (5)	0.15 (1.14)	0.43 (1.50)	0.12
	LDFR	9 (4)	11 (4)	0.02 (1.29)	0.63 (1.17)	0.001*
	Composite memory	—	—	0.17 (2.31)	1.07 (2.63)	0.007*

^1^CVLT-II: California Verbal Learning Test second edition; CR: cardiac rehabilitation; SDFR: short-delay free recall; LDFR: long-delay free recall.

^2^Two-tailed significance (paired sample *t*-tests) of differences in the CVLT-II SDFR and LDFR outcome *Z*-scores and composite memory *Z*-scores between baseline and follow-up in the normal and high cortisol groups.
